# Clinical Application and Influencing Factor Analysis of Metagenomic Next-Generation Sequencing (mNGS) in ICU Patients With Sepsis

**DOI:** 10.3389/fcimb.2022.905132

**Published:** 2022-07-13

**Authors:** Limin Sun, Shuguang Zhang, Ziyue Yang, Fei Yang, Zhenhua Wang, Hongqiang Li, Yaoguang Li, Tongwen Sun

**Affiliations:** ^1^ General Intensive Care Unit, Zhengzhou Key Laboratory of Sepsis, Henan Key Laboratory of Critical Care Medicine, The First Affiliated Hospital of Zhengzhou University, Zhengzhou, China; ^2^ Department of Infectious Diseases, The First Affiliated Hospital of Zhengzhou University, Zhengzhou, China; ^3^ Gene Hospital of Henan Province, Precision Medicine Center, The First Affiliated Hospital of Zhengzhou University, Zhengzhou, China

**Keywords:** severe infection, sepsis, microorganism culture, mNGS, NGS, intensive care unit

## Abstract

**Objective:**

To analyze the clinical application and related influencing factors of metagenomic next-generation sequencing (mNGS) in patients with sepsis in intensive care unit (ICU).

**Methods:**

The study included 124 patients with severe sepsis admitted to the ICU in the First Affiliated Hospital of Zhengzhou University from June 2020 to September 2021. Two experienced clinicians took blood mNGS and routine blood cultures of patients meeting the sepsis diagnostic criteria within 24 hours after sepsis was considered, and collection the general clinical data.

**Results:**

mNGS positive rate was higher than traditional blood culture (67.74% vs. 19.35%). APACHE II score [odds ratio (*OR*)=1.096], immune-related diseases (*OR*=6.544), and hypertension (*OR*=2.819) were considered as positive independent factors for mNGS or culture-positive. The sequence number of microorganisms and pathogen detection (mNGS) type had no effect on prognosis. Age (*OR*=1.016), female (*OR*=5.963), myoglobin (*OR*=1.005), and positive virus result (*OR*=8.531) were independent risk factors of sepsis mortality. Adjusting antibiotics according to mNGS results, there was no statistical difference in the prognosis of patients with sepsis.

**Conclusion:**

mNGS has the advantages of rapid and high positive rate in the detection of pathogens in patients with severe sepsis. Patients with high APACHE II score, immune-related diseases, and hypertension are more likely to obtain positive mNGS results. The effect of adjusting antibiotics according to mNGS results on the prognosis of sepsis needs to be further evaluated.

## Introduction

Sepsis, a major public health problem, is a syndrome physiological, pathological, and biochemical abnormality caused by infection (Singer et al., 2016). About 1.94–31.5 million deaths occur annually worldwide, of which about 20% (5.3 million deaths) are sepsis-related (Fleischmann et al., 2016; Maraki et al., 2016; Singer et al., 2016; Rudd et al., 2020). Sepsis is also the leading cause of admission to an intensive care unit (ICU) and death (Abe et al., 2018). The common infection sites include lung, abdomen, blood, urinary tracts, and central nervous system. Severe sepsis requires that the infection site, microbial species, and empirical anti-infection treatment are identified first (Rhodes et al., 2017; Cecconi et al., 2018; Niederman et al., 2021). In sepsis management strategies, early recognition and standardized management such as fluid resuscitation and hormone use are proposed, but active antibiotic use remains the cornerstone of successful sepsis treatment (Rhodes et al., 2017; Cendejas-Bueno et al., 2019; Niederman et al., 2021). Pathogen identification and beginning or adjusting the antibiotic therapy as soon as possible are essential. A traditional bacterial culture takes 3–5 days, and specific pathogenic bacteria are difficult to culture, take a longer time, have a low positive rate, difficult to diagnose, and have ineffective and delayed empirical treatment.

Metagenomic next-generation sequencing (mNGS) is a new method that combines high-throughput sequencing with bioinformatics analysis; its advantages include shorter detection time of microorganisms, accurate detection of multiple pathogens such as bacteria, fungi, viruses, and parasites at one time through DNA or RNA gene sequencing of clinical samples (Church et al., 2020; Vandenberg et al., 2020; Evans et al., 2021). This method provides a clear etiological basis for severely infected patients, and allows more targeted medication. However, as a new detection method, it is expensive with unclear clinical characteristics and influencing factors in ICU and there are few relevant studies. Thus, this study analyzed and discussed the clinical characteristics and influencing factors of mNGS in ICU.

## Materials and Methods

### Study Subjects

The study involved 199 patients admitted to the ICU of the First Affiliated Hospital of Zhengzhou University for sepsis from June 2020 to September 2021. According to the exclusion criteria and inclusion criteria, we excluded the 38 cases cerebrospinal fluid samples and 19 cases alveolar lavage fluid specimens, 5 cases of incomplete information, 8 cases with lost follow-up loss, and 5 cases with blood culture and mNGS were not conducted at the same time. Finally this study selected 124 patients admitted to the ICU of the First Affiliated Hospital of Zhengzhou University for sepsis from June 2020 to September 2021.

### Research Methods

**Inclusion criteria:** (1) It was in line with the sepsis 3.0 diagnostic criteria (Singer et al., 2016) jointly issued by the Society of Intensive Care Medicine (SCCM) and the European Society of intensive care Medicine (ESICM). (2) Agreed to mNGS sequencing for inspection. (3) Routine blood culture and mNGS were tested at the same time.

**Exclusion criteria:** (1) Did not agree to take the mNGS detection. (2) Unqualified specimens and incomplete clinical data. (3) Blood cultures and mNGS were not tested simultaneously. (4)Unknown prognosis within 28 days.

The study was approved by the Scientific Research and Clinical Trials Ethics Committee of the First Affiliated Hospital of Zhengzhou University (code 2021-KY-0600-002).

### Sample Collection

Two experienced clinicians selected patients who met the diagnostic criteria for sepsis and sent mNGS and routine blood cultures simultaneously within 24 hours after sepsis was considered.

The collected mNGS blood samples met the requirements of the First Affiliated Hospital of Zhengzhou University, Gene Hospital of Henan Province (using a special free nucleic acid tube, avoids hemolysis and strict control of the qualified specimens) and were immediately sent for detection; if the blood samples cannot be sent immediately, they were temporarily stored at room temperature. The storage time should not exceed 24 hours, and in case the temperature was more than 37°C, ice packs were used for transport, taking care that the collected blood samples were not in direct contact with ice packs (air bubble films were used for sample packaging, ice packs and mining vessel were separated by more than 15 mm to prevent frozen blood burst, causing hemolysis). The microbial nucleic acid sequences of the samples were analyzed by high-throughput sequencing technology, and then identified by comparing with the nucleic acid sequences of the existing microorganisms in the database. The mNGS detection process included experimental operation (wet experiment) and bioinformatics analysis (dry experiment). The wet experiment comprised the following four steps: sample pretreatment, nucleic acid extraction, library construction, and computer sequencing. Bioinformatics analysis involved the following steps: data quality control, human sequence removal, identification of microbial species alignment.

Thermo Scientific culture bottles was used for routine blood culture, with two sets of aerobic and anaerobic culture respectively. According to the bacteria and fungi culture procedures of Microbiology Laboratory of the First Affiliated Hospital of Zhengzhou University, routine separation media were used, including blood AGAR, chocolate AGAR, and Mueller-Hinton AGAR. Chocolate AGAR and blood AGAR plates were incubated at 5%CO_2_ at 37°C for 18–24 h. Vitek-2 Compact Instrument was used to identify the strains.

### Collection of Clinical Data

The clinical data collection of the selected subjects included: gender, age, and past medical history (hematological system, rheumatic immune system, and neoplastic diseases were classified as immune-related diseases in this study); surgical operation or not; Acute physiology and chronic health score (APACHE II); blood routine examination, procalcitonin (PCT), C-reactive protein (CRP), biochemical indicators including aspartate aminotransferase (AST), creatinine, brain natriuretic peptide (BNP), myoglobin, cholinesterase, fibrinogen (FIB) coagulative time; whether there was tracheotomy, dialysis treatment, vasopressor drug use; temperature details (greater than 38.5 °C or not); experiential antibiotic use after admission; general bacterial culture results, mNGS bacteria and sequence number results were recorded. The main observation index of clinical efficacy was the mortality rate of patients at 28 days.

### Statistical Treatment

All data in the study were statistically analyzed by SPSS23.0 and plotted using Graph Pad7.0. The Kolmogorov–Smirnov method was used for normality tests, with *P*>0.1 indicating a normal data distribution. Continuous variables with normal distribution were represented by mean ± standard deviation (mean ± SD), and data were compared by independent sample *t*-test. Non-normally distributed data were represented by median [interquartile range](median [IQR]), and data comparisons were performed using the Mann–Whitney U test. The measurement data were analyzed by Pearson’s chi-square test or Fisher’s exact test. The Kruskal–Wallis rank-sum test was used for measurement data with more than one group of non-normal distribution. Multivariate analysis was performed by binary logistic regression. In the multi-factor analysis, we selected the factors screened in the single factor analysis (variables with *P*<0.2) and then used the Enter method in logistic regression to adjust confounding factors. In this study, *P*<0.05 was considered to be statistically significant.

## Results

### Patient Baseline Characteristics

**Table 1** shows the distribution of biochemical indicators, clinical features, and past medical history.

**Table 1:** Biochemical indicators, clinical features, and past medical history.

Patient characteristic	All patients (*N*=124)
Age	56.50 (32.00,70.00)
Female	43 (34.68%)
Fever	90 (72.58%)
Heart rate	87.00 (80.00,94.00)
**Medical history**	
immune-related diseases	30 (24.19%)
Coronary heart disease	17 (13.71%)
Hypertension	48 (38.71%)
Diabetes	24 (19.35%)
Surgery	64 (51.61%)
**Disease and severity assessment scores**	
Admission APACHE II score	23.00 (18.00,23.00)
** Biochemical indicators**	
WBC	8.30 (6.23,15.30)
Procalcitonin	16.00 (3.60,100.00)
C-reactive protein	160.00 (150.00,170.00)
Platelets	67.00 (10.00,171.00)
Neutrophil%	75.20 (12.30,89.90)
Aspartate	25.00 (12.00,43.00)
Bilirubin	16.35 (8.60,80.60)
Creatinine	136.00 (42.00,154.00)
Myoglobin	406.00 (112.00,700.00)
Cholinesterase	3.20 (2.00,5.50)
Brain natriuretic peptide	2084.00 (501.00,3195.00)
Prothrombin time	16.50 (12.50,18.60)
Fibrinogen	6.56 (4.98,12.97)
**ICU treatment**	
Use of vasoactive drugs	105 (84.68%)
Hemodialysis	24 (19.35%)
Tracheotomy	103 (83.06%)
Use of Ventilator	93(75%)
28-day mortality	49 (39.52%)

WBC represents white blood count.

After screening, 124 sepsis patients with a median age of 56 years were enrolled in this study, including 43 females (34.68%) and 81 males (65.32%). In this study, 84.68% of patients had used vasoactive drugs, 83.06% of patients had received tracheotomy, 24.19% of patients had immune-related diseases, the 28-day mortality rate was 39.52%, and 107 (86.29%) of the patients underwent treatment protocol change during the treatment.

### Clinical Diagnostic Effect of mNGS and Blood Culture

**Figure 1:**
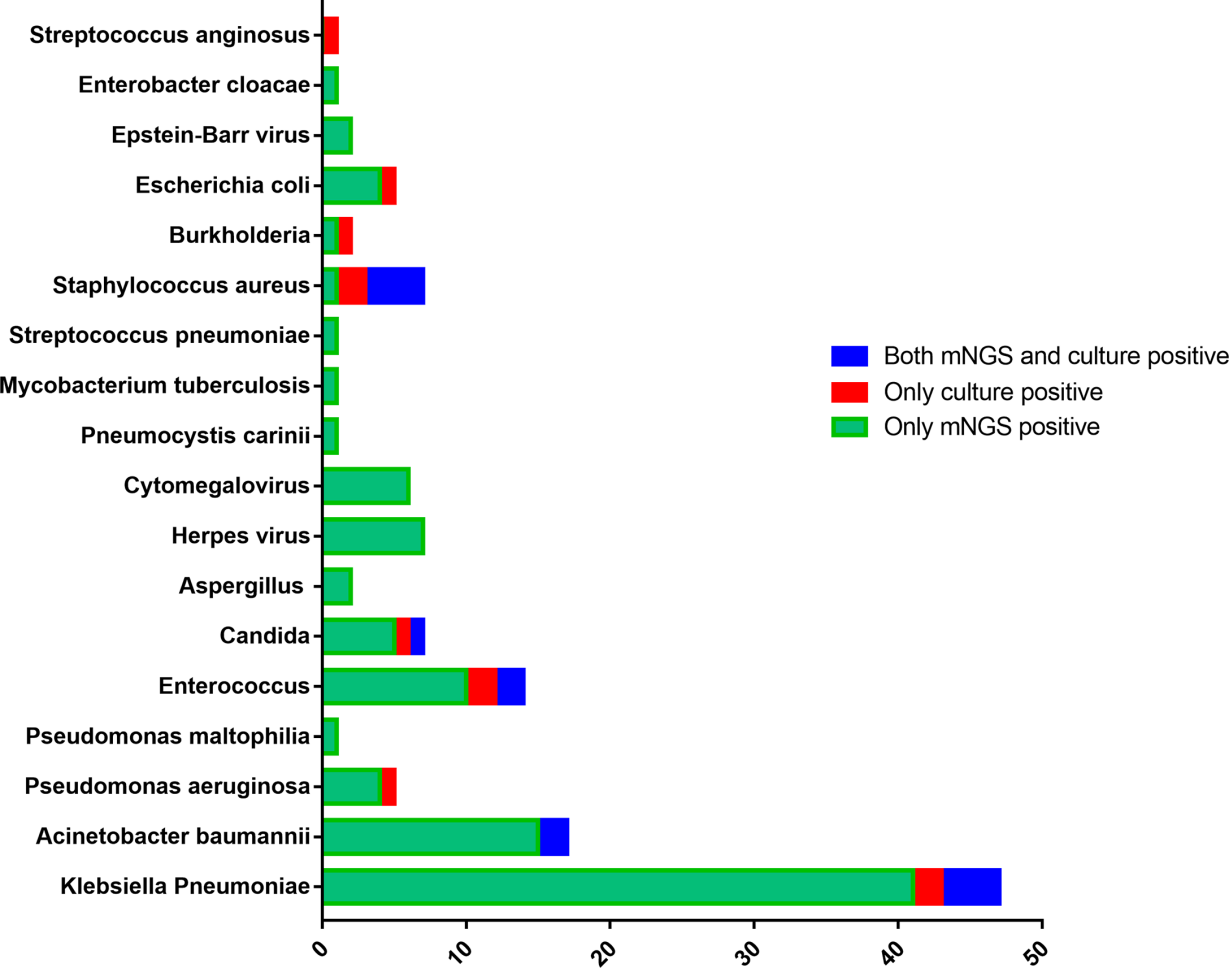
Different pathogenic organisms (in numbers) in mNGS and microbial blood cultures.

According to **Figure 1**, the top five microorganisms in the mNGS culture were: *Klebsiella pneumoniae* (*N*=41), *Acinetobacter baumannii* (*N*=17), *Enterococcus* (*N*=12), *Herpes* virus (*N*=7), *Candida* (*N*=6), and *Cytomegalovirus* (*N*=6). Similarly, in blood culture, the top five microorganisms were: *Klebsiella pneumoniae* (*N*=6), *Staphylococcus aureus* (*N*=6), *Enterococcus* (*N*=4), *Candida* (*N*=2), *Acinetobacter baumannii* (*N*=2). Thus, the top five microorganisms were *Klebsiella pneumoniae* (*N*=47), *Acinetobacter baumannii* (*N*=17) (mNGS and culture were positive in 2 patients, mNGS positive only in 15 patients), *Enterococcus* (*N*=14), *Candida* (*N*=7), *Herpes* virus (*N*=7), *Staphylococcus aureus* (*N*=7). The results also showed that the positive rate of fungal microorganisms detected by the mNGS was higher than that detected by the blood culture (7.26% VS 1.61%). 6 cases of *Candida*, 2 cases of *Aspergillus*, and 1 case of *Pneumocystis* were detected by the mNGS, and the difference was statistically significant (χ^2 =^ 4.783, *P*=0.02).

**Figure 2:**
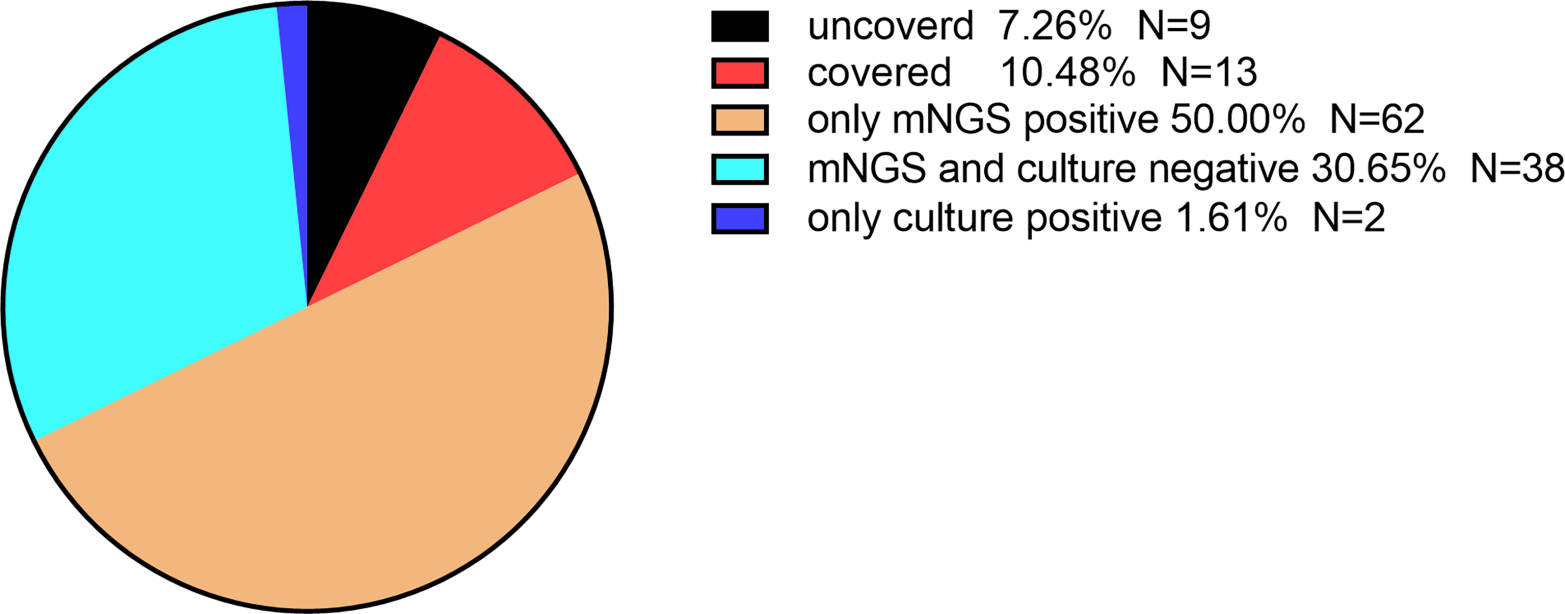
Consistency analysis of mNGS and blood culture results. *Patients, covered or uncovered, had positive mNGS and blood culture results*.

Among the 124 patients of the study, approximately 30.65% were negative for mNGS and blood cultures, and mNGS was only positive in 50% of the patients (**Figure 2**). The mNGS positive rate was higher than that of the blood culture (67.74% vs. 19.35%), and the difference was statistically significant (χ^2 =^ 59.048, *P*<0.001). The mNGS pathogen coverage rate accounted 54.17% of the total number of positive blood culture results (13/24); only two patients had positive blood culture results.

### Comparison of Negative mNGS Results With Positive mNGS Results of a Single Pathogen Infection and a Mixed Infection on the Clinical Characteristics

We further analyzed the relationship between the detection results and clinical characteristics of patients based on the mNGS results. The negative rate of mNGS and the detection rate of a single pathogen of mNGS were statistically different in sepsis patients suffering from immune-related diseases. Other biochemical indicators and clinical features had no statistically significant differences.(**Table 2**).

**Table 2:** Comparison of negative mNGS results with positive mNGS results on the clinical characteristics of a single pathogen infection and a mixed infection.

mNGS results and clinical characteristics of patients	Negative (*N*=40)	Single pathogen infection (*N*=69)	Mixed infection (*N*=15)	χ^2^/*H*	*P*
Female	16 (40%)	19	8	4.358	0.113
Age	56.00 (51.00,66.00)	58.00 (51.00,66.00)	68.00 (40.00,80.50)	1.363	0.506
Fever	27(67.50%)	50(72.46%)	13(86.67%)	2.015	0.365
Heart rate	92.00 (84.50,119.00)	102.00 (89.00,120.00)	112.00 (96.50,119.50)	2.068	0.356
**Medical history**					
immune-related diseases	4 (10.00%)*	22 (31.88%)*	4 (26.67%)	6.669	0.033
Surgery	22 (55.00%)	33 (47.83%)	9 (60%)	2.315	0.901
Coronary heart disease	6 (15.00%)	8 (11.59%)	3 (20.00%)	0.819	0.662
Hypertension	11 (27.50%)	32 (46.38%)	5 (33.33%)	4.011	0.135
Diabetes	5 (12.50%)	16 (23.19%)	3 (20.00%)	1.858	0.417
**Disease and severity assessment scores**					
Admission APACHE II score	17.50 (16.50,19.50)	18.00 (16.00,23.00)	18.00 (16.00,20.50)	1.656	0.437
**Biochemical indicators**					
WBC	11.58 (9.14,13.20)	11.14 (6.40,17.20)	10.10 (3.20,17.80)	0.045	0.978
Procalcitonin	3.40 (0.36,17.00)	1.19 (0.52,8.06)	1.20 (0.71,5.55)	0.081	0.961
C-reactive protein	113.00 (27.61,233.52)	76.00 (16.20,149.17)	49.00 (26.00,93.65)	1.563	0.458
Platelets	179.00 (127.00,268.00)	149.00 (86.00,239.00)	110.00 (47.00,162.00)	4.030	0.133
Neutrophil%	85.55 (73.45,90.60)	86.70 (73.10,91.90)	81.50 (61.95,90.60)	0.736	0.692
Aspartate	23.00 (17.50,72.50)	26.00 (17.00,53.00)	24.00 (19.50,40.50)	0.021	0.989
Bilirubin	12.25 (6.75,23.41)	13.92 (8.60,20.50)	10.50 (8.00,16.95)	0.703	0.704
Creatinine	91.00 (58.00,145.00)	83.00 (55.00,131.00)	65.00 (40.50,165.50)	1.417	0.492
Myoglobin	272.50 (43.18,708.05)	160.00 (45.00,553.00)	350.00 (236.15,546.65)	1.882	0.39
Cholinesterase	4.30 (2.95,7.05)	4.20 (2.70,5.80)	3.60 (2.85,4.85)	1.466	0.48
Brain natriuretic peptide	411.00 (240.00,2143.00)	969.00 (278.40,4206.00)	1667.00 (582.00,3969.50)	4.005	0.135
Prothrombin time	11.95 (10.85,15.20)	12.80 (11.40,14.70)	14.00 (11.75,14.75)	0.794	0.672
Fibrinogen	3.92 (2.78,5.01)	3.60 (2.78,4.56)	3.12 (2.39,4.23)	1.166	0.558
**ICU treatment**					
Use of Ventilator	32(80.00%)	49(71.01%)	12(80.00%)	1.318	0.517
Use of vasoactive drugs	32(80.00%)	59(85.51%)	14(93.33%)	1.577	0.454
Hemodialysis	7(17.50%)	14(20.29%)	3(20.00%)	0.131	0.937
Tracheotomy	36(90.00%)	53(76.81%)	14(93.33%)	4.41	0.11
28-day mortality	16(40.00%)	24(34.78%)	9(60.00%)	3.284	0.194

*Compared with patients without immune-related diseases, there was a statistical difference, P<0.05.

### Comparison of Clinical Characteristics Between the Two Groups With Both Negative mNGS and Blood Culture Results and Positive mNGS or Blood Culture Results

Both blood culture and mNGS positive results can provide significant diagnostic and treatment values, but there were some patients with double negative results (both blood culture and mNGS showed negative results). Univariate analysis was performed in advance to identify factors associated with a positive outcome; the results showed that patients with immune-related diseases had lesser double-negative results (*P*=0.022). Additionally, the APACHE II score (*P*=0.077), platelet (*P*=0.060), BNP, and hypertension (*P*=0.073) were close to statistical significance. There were no statistically significant differences between other biochemical indicators and clinical features (**Table 3**).

**Table 3:** Comparison of clinical characteristics between mNGS and blood culture (positive and negative).

	mNGS and culture-negative (*N*=38)	mNGS or culture-positive (*N*=86)	χ^2^/U	*P*
Age	56.00 (51.00,68.00)	58.00 (51.00,67.00)	1526.000	0.558
Female	14 (36.84%)	29 (33.72%)	0.113	0.838
Fever	12 (31.58%)	22 (25.58%)	0.476	0.517
Heart rate	92.00 (84.00,120.00)	102.50 (90.00,120.00)	1432.000	0.273
**Medical history**				
immune-related diseases	4 (10.53%)	26 (30.23%)	5.580	0.022
Coronary heart disease	6 (15.79%)	11 (12.79%)	0.200	0.778
Hypertension	10 (26.32%)	38 (44.19%)	3.547	0.073
Diabetes	4 (10.53%)	20 (23.26%)	2.736	0.139
Surgery	16 (42.11%)	44 (51.16%)	0.866	0.436
**Disease and severity assessment scores**				
Admission APACHE II score	17.00 (16.00,19.00)	18.00 (16.00,23.00)	1309.000	0.077
**Biochemical indicators**				
WBC	11.58 (8.87,13.20)	10.48 (6.31,17.34)	1589.000	0.807
Procalcitonin	3.40 (0.36,17.00)	1.20 (0.57,8.06)	1590.000	0.812
C-reactive protein	113.00 (27.61,249.10)	74.50 (16.20,149.17)	1419.000	0.244
Platelets	179.00 (127.00,268.00)	145.50 (65.00,223.00)	1287.000	0.060
Neutrophil%	84.30 (71.50,90.60)	86.75 (73.10,91.90)	1533.000	0.584
Aspartate	23.00 (16.00,50.00)	26.50 (17.00,54.00)	1539.000	0.606
Bilirubin	11.90 (6.50,21.70)	13.86 (8.50,20.50)	1443.000	0.301
Creatinine	91.00 (58.00,145.00)	78.50 (54.00,134.00)	1440.000	0.293
Myoglobin	272.50 (43.04,707.50)	228.15 (60.00,553.30)	1623.500	0.955
Cholinesterase	4.20 (2.90,7.00)	4.10 (2.70,5.80)	1530.000	0.573
Brain natriuretic peptide	411.00 (240.00,2202.00)	1026.00 (278.40,4206.00)	1316.000	0.085
Prothrombin time	11.70 (10.80,15.30)	12.85 (11.40,14.80)	1444.000	0.303
Fibrinogen	3.92 (2.83,5.06)	3.46 (2.50,4.50)	1415.500	0.236
**ICU treatment**				
Use of vasoactive drugs	30(78.95%)	75(87.21%)	1.387	0.239
Hemodialysis	32 (84.21%)	68 (79.07%)	0.446	0.625
Tracheotomy	34 (89.47%)	69 (80.23%)	1.600	0.300
Use of Ventilator	30(78.95%)	63(73.26%)	0.455	0.500
28-day mortality	14 (36.84%)	35 (40.70%)	0.164	0.697

Next, we conducted multivariate analysis by binary logistic regression for *P*<0.2 in univariate analysis, which showed that the APACHE II score (*OR*=1.096), immune-related diseases (*OR*=6.544), hypertension (*OR*=2.819) were considered independent factors; the higher the indicators, the higher was the positive rate of the mNGS or blood culture. Diabetes had a tendency to be an independent factor affecting positive mNGS or blood culture results (*OR*=3.208, *P*=0.070) (**Table 4**).

**Table 4:** Risk factors for mNGS or culture – positive.

	*OR*	95%*CI*	*P* value
Admission APACHE II score	1.096	(1.006 – 1.195)	0.036
Platelets	1.001	(0.998 – 1.004)	0.552
Brain natriuretic peptide	1.000	(1.000 – 1.000)	0.153
Underlying diseases that affect immunity	6.544	(1.835 – 23.335)	0.004
Hypertension	3.413	(1.069 – 7.431)	0.036
Diabetes	3.208	(0.909 – 11.320)	0.070

### Analysis of Factors Affecting the Prognosis of Sepsis Patients

Sepsis patients have poor prognoses, so we included routine biochemical indicators and clinical features of the patients for analysis along with mNGS results for analysis (**Table 5**). The univariate analysis determined the indicators of *P*<0.2 (age, women, surgery, APACHE II score at admission, platelet, percentage of neutrophils, myoglobin, BNP, heart rate, mNGS results positive virus, and immune-related diseases); other indicators did not follow the next multivariate analysis. In univariate analysis, the sequence number of microorganisms and pathogen detection (mNGS) type such as negative, infection with a single pathogen and mixed infection had no effect on prognosis. Further binary logistic regression analysis of *P*<0.2 above (**Table 6**) suggested that age (*OR*=1.061), female (*OR*=5.963), myoglobin (*OR*=1.005), and positive viral results (*OR*=8.531) were independent risk factors.

**Table 5:** Analysis of factors affecting the prognosis of sepsis patients.

	Survivors (*N*=75)	Non-survivors (*N*=49)	χ^2^/U	*P*
Age	56.00 (51.00,62.00)	66.00 (51.00,75.00)	1300.000	0.006
Female	19 (33.93%)	24 (48.98%)	7.316	0.007
Fever	52 (69.33%)	38 (77.56%)	1.006	0.316
Heart rate	101.00 (85.00,118.00)	102.00 (90.00,120.00)	1561.500	0.158
**Medical history**				
immune-related diseases	14 (18.67%)	16 (32.65%)	3.161	0.075
Coronary heart disease	11 (14.67%)	6 (12.25%)	0.147	0.701
Hypertension	30 (40.00%)	18 (36.74%)	0.133	0.715
Diabetes	13 (17.33%)	11 (22.45%)	0.497	0.481
Hemodialysis	15 (20.00%)	9(18.37%)	0.051	0.822
Surgery	32 (42.67%)	28 (57.14%)	2.487	0.115
**Disease and severity assessment scores**				
Admission APACHE II score	18 (13,21)	18 (17,21)	1551.000	0.141
**Biochemical indicators**				
WBC	11.14 (8.355,14.465)	10.26 (3.64,17)	1709.000	0.511
Procalcitonin	1.19 (0.39,11.35)	3.6(0.57,10.00)	1747.000	0.644
C-reactive protein	73.20 (10.80,145.09)	88.47 (28.00,162.45)	1622.000	0.271
Platelets	179.00 (123.5.00,247.50)	114.00 (30.00,193.00)	1204.000	0.001
Neutrophil%	87.00 (78.85,90.85)	84.20 (64.80,92.30)	1552.500	0.145
Aspartate	29.00 (16.50,56.50)	23.00 (17.00,47.00)	1642.500	0.319
Bilirubin	11.90 (6.95,19.55)	13.92 (10.00,21.30)	1611.000	0.247
Creatinine	80.00 (55.00,124.50)	85.00 (57.10,156.00)	1588.500	0.203
Myoglobin	70.75 (37.29,251.15)	700.00 (350.50,1052.00)	410.500	<0.001
Cholinesterase	4.20 (3.00,6.45)	3.90 (2.50,6.00)	1651.000	0.340
Brain natriuretic peptide	549.00 (161.30,3636.00)	1063.00 (411.00,3713.00)	1511.500	0.096
Prothrombin time	12.60 (11.40,14.70)	12.70 (11.20,15.30)	1781.000	0.773
Fibrinogen	3.70 (2.82,5.02)	3.45 (2.52,4.42)	1650.000	0.338
**ICU treatment**				
Use of vasoactive drugs	63 (84.00%)	42 (85.71%)	0.067	0.796
Hemodialysis	15 (20.00%)	9(18.37%)	0.051	0.822
Tracheotomy	63 (81.33%)	42 (85.71%)	0.404	0.525
Virus positive (mNGS)	6 (8.00%)	8(16.3%)	2.052	0.152
Sequence number				
Negative	24 (32.00%)	16 (32.65%)	6.817	0.227
0–50	27 (36.00%)	9 (18.37%)		
51–100	6 (8.00%)	4 (8.16%)		
100–500	9 (12.00%)	9 (18.37%)		
500–1000	3 (4.00%)	2 (4.08%)		
>1000	6 (8.00%)	9 (18.37%)		
Pathogen detection (mNGS)				
Negative	24 (32.00%)	16 (32.65%)	3.284	0.194
Infection with a single pathogen	45 (60.00%)	24 (48.98%)		
Mixed infection	6 (8.00%)	9 (18.37%)		
Blood culture positive	15 (20.00%)	9 (18.37%)	0.051	1.000

**Table 6:** Risk factors for mortality.

	*OR*	95%*CI*	*P*
Age	1.061	(1.015 – 1.109)	0.009
Female	5.963	(1.579 – 22.518)	0.008
Underlying diseases that affect immunity	0.585	(0.123 – 2.769)	0.499
Admission APACHE II score	1.097	(0.996 – 1.207)	0.060
Neutrophil%	0.946	(0.914 – 0.980)	0.002
Myoglobin	1.005	(1.003 – 1.007)	<0.001
Brain natriuretic peptide	1.000	(1.000 – 1.000)	0.344
Heart rate	1.001	(0.970 – 1.033)	0.956
Surgery	3.612	(0.982 – 13.283)	0.053
Virus positive	8.531	(1.277 – 57.007)	0.027

### Comparison of Antimicrobial Adjustment for Outcomes in Sepsis Patients According to mNGS Results and Blood Culture Results

This study analyzed the prognosis of patients with positive mNGS results and adjusted or not adjusted treatment regimens. Seventy-seven patients were adjusted and seven patients were not adjusted; there was no statistical difference in survival rate between the two groups (59.74% vs. 71.42%, χ^2 =^ 0.368, *P*=0.544).

Patients with negative mNGS results, with 30 and 10 patients in the regimen-adjusted and unadjusted groups, respectively, had a survival rate of 60% in both groups, (χ^2 =^ 0.000, *P*=1.000).

## Discussion

Severe infections in ICU patients progress and change rapidly. Sepsis guidelines (Cecconi et al., 2018; Dugar et al., 2020) recommend identifying the infection site first, followed by collecting the body-fluid specimens for routine culture, identifying pathogens, and finally, selecting the targeted antimicrobial drugs. The correct use of antimicrobial agents in the early stages is critical for the prognosis of patients with sepsis or septic shock. However, the positive rate of a traditional blood culture is about 20% lower and about 3–5 days longer (Greninger and Naccache, 2019). For particular bacteria cultures, *Mycobacterium* tuberculosis needs a longer culture time, and the positive rate of pathogens such as *Nocardia*, *Cryptococcus*, and *Brucella* is lower, while viruses fail to culture (Mancini et al., 2010; Siwakoti et al., 2018; Zhang H. C., et al., 2019; Li et al., 2021), thus causing a delay in the treatment. The mNGS can be obtained within 24 hours, has unique advantages for the traditional bacterial culture, and helps screen the pathogenic bacteria, thereby guiding the clinical application better. We discussed the clinical characteristics and value of its function as a new pathogen test in patients with severe sepsis in ICU.

A total of 124 patients with severe sepsis were included in this study, most of whom received vasoactive drugs, had a high APACHE II score, undergone tracheotomy and transferred from sub-hospitals. According to the pathogens found: G-bacillus accounted for the highest proportion; the top two pathogens were *Klebsiella pneumoniae*, *Acinetobacter baumannii*, which was consistent with the current epidemiology of hospital-related infections (Kaye and Pogue, 2015; Dugar et al., 2020; Geng et al., 2021). Additionally, one case of *Pneumocystis* and one tuberculosis pathogens were found. *Pneumocystis jirovecii* was found in mNGS of a patient with a malignant hematologic tumor complicated with septic shock. Related studies have also shown that although pneumocystis is almost exclusively present in the human lung, pneumocystis fragments can enter the peripheral blood through the site of respiratory infection, especially under immunosuppression (Zhang Y., et al., 2019; Chen et al., 2020; Zhang et al., 2021). It suggests that blood mNGS can help diagnose *Pneumocystis jirovecii*, which is difficult to diagnosed. In another case of immune-related disease, fragments of bacterium tuberculosis was found in the blood and led to septic shock. This was very challenging for the clinician to determine and distinguish (Mishra et al., 2019; Sun et al., 2021). The mNGS fungi detection rate was significantly higher than the blood culture results with significant statistical differences, and virus detection was impossible in the blood culture. This is consistent with the views of Zheng et al. (Zheng et al., 2022). Highlighting the clinical advantages of NGS in finding particular fastidious pathogenic bacteria that can guide the clinical treatment better (Kruppa et al., 2018; Duan et al., 2021b; Govender et al., 2021; Tsang et al., 2021; Zhan et al., 2022).

This study showed that the mNGS positive rate was higher, and the mNGS pathogen coverage accounted for 54.17% (13/24) of the total number of positive blood culture results, and two other patients showed positive results only in blood cultures. This indicates that mNGS may miss pathogens, be inconsistent with blood culture, have false negative results, and need for higher sequencing depth detection (Duan et al., 2021a; Gu W., et al., 2021). Natoli et al. (2022) also agreed that any contamination with the blood culture between sample processing and data analysis could bias the final results seriously. Boers et al. (Boers et al., 2019) also showed that the above reasons lead to the discovery of non-existent bacterial genera, false correlations between microbes and their hosts, and the inability to detect true correlations. However,The advantages of mNGS detection were not affected by history of antimicrobial exposure. In 2019, Blauwkamp et al. (Blauwkamp et al., 2019) found that microbial cell-free DNA (mcfDNA) sequencing test performed much better than blood culture in analyzing specimens from subjects who had received antimicrobial therapy within two weeks preceding presentation. The reason is that microbial sequencing methods diagnose possible infections by capturing and identifying this highly fragmented mcfDNA in the circulating system, which are lysed fragments of bacteria and conventional blood culture failed to detect (Han et al., 2020).

Our study reported that mNGS showed a higher positive rate in patients having immune-related diseases with sepsis, and a statistically significant higher detection rate of single pathogen infection compared to the no immune-related diseases in patients with sepsis. The earlier studys (Geng et al., 2021; Yan et al., 2021) showed that low immunity was associated with multiple infections. Niles et al., (Niles et al., 2022) in a retrospective study of 169 participants, showed that immune-related patients were more likely to obtain multiple microbial results from mNGS than patients with normal immune function.

Subsequently, we analyzed the influence of sepsis patients with negative culture and mNGS results and positive results of mNGS or blood culture bacteriology. Univariate analysis and multivariate analysis suggesting that sepsis patients with high APACHE II score, immune-related diseases, hypertension had lower double-negative results. This means the more severe the disease, the easier it is to obtain positive blood cultures or mNGS results. There were few reports on the clinical influencing factors of mNGS results. As far as we know, only one study shows that age is a significant influencing factor in the multi-factor logic analysis of positive mNGS results (Duan et al., 2021a).

We included mNGS reads in univariate and multivariate analyses of its impact on the prognosis of patients with sepsis. Our research found that microbial sequence number and pathogen detection (mNGS) type had no direct relationship to the prognosis of sepsis. And age, female, myoglobin and virus-positive results were independent risk factors for sepsis. This suggests that mNGS reads represent the presence of certain bacterial infections, and cannot be associated with disease prognosis. Ong et al. (Ong et al., 2017) showed in a prospective study center that 68% of the septic shock patients activated after viral infection without prior immune deficiency were independently associated risk factors for sepsis mortality. Age, female, and myoglobin are more analyzed in the influencing factors of sepsis (Yang et al., 2019; Gu B., et al., 2021).

This study showed no statistical difference in the overall prognosis of sepsis according to mNGS adjustment regimen, and this may be related to the source of patients, severe septic shock, and small sample size. However, there is no doubt that mNGS can be used to quickly, and a high positive rate to obtain pathogens included fastidious bacteria (Huang et al., 2019). The results of Geng et al. (Geng et al., 2021) were in agreement with the above-mentioned results. mNGS has obvious advantages as it can quickly, efficiently, and accurately obtain all nucleic acid information in test samples, analyze pathogens, guide clinical diagnosis and treatment, and find viruses, fungi, parasites, rare pathogens, and even unknown pathogens (Consensus Group Of Experts On Application Of Metagenomic Next Generation Sequencing In The Pathogen Diagnosis In Clinical et al., 2020; Han, 2022). Zhou et al. (Zhou et al., 2021) described the use of mNGS for pneumonia pathogen identification in a large-scale multi-center prospective study of 159 patients, which resulted in 59 patients (37.1%) changing treatment regimens, including 40 patients (25.2%) downgrading antibiotic use. There are few studies on the application of mNGS in ICU patients, so it is necessary to expand the sample size and further explore the effect of adjusting antibacterial application according to the mNGS results on the prognosis of ICU patients with sepsis.

This study has some limitations. First of all, the origin of the patients (transfered from subordinate hospitals and in a critical condition) may cause negative mNGS and blood culture results. In addition, the antibiotic exposure history has a greater impact on blood culture, leading to an increase in the negative rate of blood culture. Secondly, our sample size is small. Therefore, further large-sample studies are needed to explore the application of mNGS in sepsis. Thirdly, the study does not represent the application characteristics of mNGS in other infections, fever and pathogenic bacteria in difficult cases, and in critically ill patients in other regions.

In conclusion, mNGS can quickly obtain pathogenic bacteria (including fastidious bacteria), have a higher positive rate. The mNGS positive rate was higher than that of the blood culture, but there are still some negative results. Patients with high APACHE II score, immune-related diseases, hypertension had lower double-negative results. The sequence number of microorganisms and pathogen detection (mNGS) type such as negative,infection with a single pathogen and mixed infection were not directly related to the disease prognosis. There was no statistical difference in prognosis of sepsis according to mNGS adjustment regimen. However, mNGS is very important for the acquisition of pathogenic bacteria in severe patients, and we would carry out a larger sample study to explore the impact of mNGS on the outcome of sepsis bloodstream infection.

## Data Availability Statement

The data presented in the study are deposited in the ERP repository, accession number PRJEB53676.

## Ethics Statement

The studies involving human participants were reviewed and approved by The study was approved by the Scientific Research and Clinical Trials Ethics Committee of the First Affiliated Hospital of Zhengzhou University (code 2021-KY-0600-002). The patients/participants provided their written informed consent to participate in this study.

## Author Contributions

LMS and SGZ were responsible for managing the patient. ZYY and FY collected data. ZHW, HQL, and TWS revised the article. All authors have read and approved the final manuscript and contributed substantially to the work presented in this article.

## Funding

This study was supported by the 51282 Project Leaders of Scientific and Technological Innovative Talents from Health and Family Planning Commission in Henan Province (2016-32), and Science and Technology people-benefit project of Zhengzhou (2019KJHM0001).

## Conflict of Interest

The authors declare that the research was conducted in the absence of any commercial or financial relationships that could be construed as a potential conflict of interest.

## Publisher’s Note

All claims expressed in this article are solely those of the authors and do not necessarily represent those of their affiliated organizations, or those of the publisher, the editors and the reviewers. Any product that may be evaluated in this article, or claim that may be made by its manufacturer, is not guaranteed or endorsed by the publisher.

## Abbreviations

mNGS, metagenomic next-generation sequencing; ICU, intensive care unit; OR, odds ratio; SCCM, Society of Intensive Care Medicine; ESICM, European Society of intensive care Medicine; APACHE II, Acute physiology and chronic health score; PCT, procalcitonin; CRP, C-reactive protein; AST, aspartate aminotransferase; BNP, brain natriuretic peptide; FIB, fibrinogen; SD, standard deviation; IQR, interquartile range.

## References

[B1] Abe T. Ogura H. Shiraishi A. Kushimoto S. Saitoh D. Fujishima S.. (2018). Characteristics, Management, and in-Hospital Mortality Among Patients With Severe Sepsis in Intensive Care Units in Japan: The FORECAST Study. Crit. Care 22 (1), 322. doi: 10.1186/s13054-018-2186-7 30466493 PMC6251147

[B2] Blauwkamp T. A. Thair S. Rosen M. J. Blair L. Lindner M. S. Vilfan I. D.. (2019). Analytical and Clinical Validation of a Microbial Cell-Free DNA Sequencing Test for Infectious Disease. Nat. Microbiol. 4, 663–674. doi: 10.1038/s41564-018-0349-6 30742071

[B3] Boers S. A. Jansen R. Hays J. P. (2019). Understanding and Overcoming the Pitfalls and Biases of Next-Generation Sequencing (NGS) Methods for Use in the Routine Clinical Microbiological Diagnostic Laboratory. Eur. J. Clin. Microbiol. Infect. Dis. 38 (6), 1059–1070. doi: 10.1007/s10096-019-03520-3 30834996 PMC6520317

[B4] Cecconi M. Evans L. Levy M. Rhodes A. (2018). Sepsis and Septic Shock. Lancet 392 (10141), 75–87. doi: 10.1016/s0140-6736(18)30696-2 29937192

[B5] Cendejas-Bueno E. Romero-Gómez M. P. Mingorance J. (2019). The Challenge of Molecular Diagnosis of Bloodstream Infections. World J. Microbiol. Biotechnol. 35 (4), 65. doi: 10.1007/s11274-019-2640-y 30941578

[B6] Chen J. He T. Li X. Wang X. Peng L. Ma L.. (2020). Metagenomic Next-Generation Sequencing in Diagnosis of a Case of Pneumocystis Jirovecii Pneumonia in a Kidney Transplant Recipient and Literature Review. Infect. Drug Resist. 13, 2829–2836. doi: 10.2147/IDR.S257587 32884306 PMC7431457

[B7] Church D. L. Cerutti L. Gürtler A. Griener T. Zelazny A. Emler S. (2020). Performance and Application of 16S rRNA Gene Cycle Sequencing for Routine Identification of Bacteria in the Clinical Microbiology Laboratory. Clin. Microbiol. Rev. 33 (4), e00053–19. doi: 10.1128/cmr.00053-19 32907806 PMC7484979

[B8] Consensus Group Of Experts On Application Of Metagenomic Next Generation Sequencing In The Pathogen Diagnosis In Clinical Professional Committee Of, S Shock Chinese Research Hospital, A Professional Committee Of Microbial Toxins Chinese Society For, M Professional Committee Of Critical Care Medicine Shenzhen Medical, A. (2020). [Expert Consensus for the Application of Metagenomic Next Generation Sequencing in the Pathogen Diagnosis in Clinical Moderate and Severe Infections (First Edition)]. Zhonghua Wei Zhong Bing Ji Jiu Yi Xue 32 (5), 531–536. doi: 10.3760/cma.j.cn121430-20200228-00095 32576342

[B9] Duan H. Li X. Mei A. Li P. Liu Y. Li X.. (2021a). The Diagnostic Value of Metagenomic Next⁃Generation Sequencing in Infectious Diseases. BMC Infect. Dis. 21 (1), 62. doi: 10.1186/s12879-020-05746-5 33435894 PMC7805029

[B10] Duan L. W. Qu J. L. Wan J. Xu Y. H. Shan Y. Wu L. X.. (2021b). Effects of Viral Infection and Microbial Diversity on Patients With Sepsis: A Retrospective Study Based on Metagenomic Next-Generation Sequencing. World J. Emerg. Med. 12 (1), 29–35. doi: 10.5847/wjem.j.1920-8642.2021.01.005 33505547 PMC7790710

[B11] Dugar S. Choudhary C. Duggal A. (2020). Sepsis and Septic Shock: Guideline-Based Management. Cleve Clin. J. Med. 87 (1), 53–64. doi: 10.3949/ccjm.87a.18143 31990655

[B12] Evans L. Rhodes A. Alhazzani W. Antonelli M. Coopersmith C. M. French C.. (2021). Surviving Sepsis Campaign: International Guidelines for Management of Sepsis and Septic Shock 2021. Intensive Care Med. 47 (11), 1181–1247. doi: 10.1007/s00134-021-06506-y 34599691 PMC8486643

[B13] Fleischmann C. Scherag A. Adhikari N. K. Hartog C. S. Tsaganos T. Schlattmann P.. (2016). Assessment of Global Incidence and Mortality of Hospital-Treated Sepsis. Current Estimates and Limitations. Am. J. Respir. Crit. Care Med. 193 (3), 259–272. doi: 10.1164/rccm.201504-0781OC 26414292

[B14] Geng S. Mei Q. Zhu C. Fang X. Yang T. Zhang L.. (2021). Metagenomic Next-Generation Sequencing Technology for Detection of Pathogens in Blood of Critically Ill Patients. Int. J. Infect. Dis. 103, 81–87. doi: 10.1016/j.ijid.2020.11.166 33227513

[B15] Govender K. N. Street T. L. Sanderson N. D. Eyre D. W. (2021). Metagenomic Sequencing as a Pathogen-Agnostic Clinical Diagnostic Tool for Infectious Diseases: A Systematic Review and Meta-Analysis of Diagnostic Test Accuracy Studies. J. Clin. Microbiol. 59 (9), e0291620. doi: 10.1128/jcm.02916-20 33910965 PMC8373000

[B16] Greninger A. L. Naccache S. N. (2019). Metagenomics to Assist in the Diagnosis of Bloodstream Infection. J. Appl. Lab. Med. 3 (4), 643–653. doi: 10.1373/jalm.2018.026120 31639732

[B17] Gu W. Deng X. Lee M. Sucu Y. D. Arevalo S. Stryke D.. (2021). Rapid Pathogen Detection by Metagenomic Next-Generation Sequencing of Infected Body Fluids. Nat. Med. 27 (1), 115–124. doi: 10.1038/s41591-020-1105-z 33169017 PMC9020267

[B18] Gu B. Liu N. Nie Y. Liu Z. M. Liu Y. J. Chen M. Y.. (2021). The Prognostic Value of Myoglobin Difference in Sepsis Related Chronic Critical Illness. Zhonghua Nei Ke Za Zhi 60 (4), 350–355. doi: 10.3760/cma.j.cn112138-20200721-00691 33765705

[B19] Han S. Y. (2022). Clinical Value of Metagenomic Next-Generation Sequencing in Complicated Infectious Diseases. Zhongguo Dang Dai Er Ke Za Zhi 24 (2), 210–215. doi: 10.7499/j.issn.1008-8830.2110064 35209988 PMC8884048

[B20] Han D. Li R. Shi J. Tan P. Zhang R. Li J. (2020). Liquid Biopsy for Infectious Diseases: A Focus on Microbial Cell-Free DNA Sequencing. Theranostics 10 (12), 5501–5513. doi: 10.7150/thno.45554 32373224 PMC7196304

[B21] Huang Z. Zhang C. Li W. Fang X. Wang Q. Xing L.. (2019). Metagenomic Next-Generation Sequencing Contribution in Identifying Prosthetic Joint Infection Due to Parvimonas Micra: A Case Report. J. Bone Jt Infect. 4 (1), 50–55. doi: 10.7150/jbji.30615 30755848 PMC6367198

[B22] Kaye K. S. Pogue J. M. (2015). Infections Caused by Resistant Gram-Negative Bacteria: Epidemiology and Management. Pharmacotherapy 35 (10), 949–962. doi: 10.1002/phar.1636 26497481

[B23] Kruppa J. Jo W. K. van der Vries E. Ludlow M. Osterhaus A. Baumgaertner W.. (2018). Virus Detection in High-Throughput Sequencing Data Without a Reference Genome of the Host. Infect. Genet. Evol. 66, 180–187. doi: 10.1016/j.meegid.2018.09.026 30292006

[B24] Li N. Cai Q. Miao Q. Song Z. Fang Y. Hu B. (2021). High-Throughput Metagenomics for Identification of Pathogens in the Clinical Settings. Small Methods 5 (1), 2000792. doi: 10.1002/smtd.202000792 33614906 PMC7883231

[B25] Mancini N. Carletti S. Ghidoli N. Cichero P. Burioni R. Clementi M. (2010). The Era of Molecular and Other Non-Culture-Based Methods in Diagnosis of Sepsis. Clin. Microbiol. Rev. 23 (1), 235–251. doi: 10.1128/cmr.00043-09 20065332 PMC2806664

[B26] Maraki S. Mantadakis E. Mavromanolaki V. E. Kofteridis D. P. Samonis G. (2016). A 5-Year Surveillance Study on Antimicrobial Resistance of Acinetobacter Baumannii Clinical Isolates From a Tertiary Greek Hospital. Infect. Chemother. 48 (3), 190–198. doi: 10.3947/ic.2016.48.3.190 27659437 PMC5048000

[B27] Mishra R. Patel H. K. Singasani R. Vakde T. (2019). Tuberculosis Septic Shock, an Elusive Pathophysiology and Hurdles in Management: A Case Report and Review of Literature. World J. Crit. Care Med. 8, 72–81. doi: 10.5492/wjccm.v8.i5.72 31559146 PMC6753395

[B28] Natoli R. M. Marinos D. P. Montalvo R. N. Degani Y. Ochenjele G. Griffith C.. (2022). Poor Agreement Between Next-Generation DNA Sequencing and Bacterial Cultures in Orthopaedic Trauma Procedures. J. Bone Joint Surg. Am. 104 (6), 497–503. doi: 10.2106/jbjs.21.00785 35041629

[B29] Niederman M. S. Baron R. M. Bouadma L. Calandra T. Daneman N. DeWaele J.. (2021). Initial Antimicrobial Management of Sepsis. Crit. Care 25 (1), 307. doi: 10.1186/s13054-021-03736-w 34446092 PMC8390082

[B30] Niles D. T. Revell P. A. Ruderfer D. Marquez L. McNeil J. C. Palazzi D. L. (2022). Clinical Impact of Plasma Metagenomic Next-Generation Sequencing in a Large Pediatric Cohort. Pediatr. Infect. Dis. J. 41 (2), 166–171. doi: 10.1097/INF.0000000000003395 34845152

[B31] Ong D. S. Y. Bonten M. J. M. Spitoni C. Verduyn, Lunel F. M. Frencken J. F. Horn J.. (2017). Epidemiology of Multiple Herpes Viremia in Previously Immunocompetent Patients With Septic Shock. Clin. Infect. Dis. 64 (9), 1204–1210. doi: 10.1093/cid/cix120 28158551

[B32] Rhodes A. Evans L. E. Alhazzani W. Levy M. M. Antonelli M. Ferrer R.. (2017). Surviving Sepsis Campaign: International Guidelines for Management of Sepsis and Septic Shock: 2016. Intensive Care Med. 43 (3), 304–377. doi: 10.1007/s00134-017-4683-6 28101605

[B33] Rudd K. E. Johnson S. C. Agesa K. M. Shackelford K. A. Tsoi D. Kievlan D. R.. (2020). Global, Regional, and National Sepsis Incidence and Mortality 1990-2017: Analysis for the Global Burden of Disease Study. Lancet 395 (10219), 200–211. doi: 10.1016/s0140-6736(19)32989-7 31954465 PMC6970225

[B34] Singer M. Deutschman C. S. Seymour C. W. Shankar-Hari M. Annane D. Bauer M.. (2016). The Third International Consensus Definitions for Sepsis and Septic Shock (Sepsis-3). Jama 315 (8), 801–810. doi: 10.1001/jama.2016.0287 26903338 PMC4968574

[B35] Siwakoti S. Subedi A. Sharma A. Baral R. Bhattarai N. R. Khanal B. (2018). Incidence and Outcomes of Multidrug-Resistant Gram-Negative Bacteria Infections in Intensive Care Unit From Nepal- a Prospective Cohort Study. Antimicrob. Resist. Infect. Control 7, 114. doi: 10.1186/s13756-018-0404-3 30275945 PMC6158849

[B36] Sun L. Yang Z. Yang F. Wang Z. Li H. Wang H.. (2021). Diagnosis of Mycobacterium Tuberculosis Septic Shock in Patients With Anti-Synthetase Syndrome Based on Next-Generation Sequencing: A Case Report and Literature Review. Front. Med. (Lausanne) 8. doi: 10.3389/fmed.2021.675041 PMC8281055 34277657

[B37] Tsang C. C. Teng J. L. L. Lau S. K. P. Woo P. C. Y. (2021). Rapid Genomic Diagnosis of Fungal Infections in the Age of Next-Generation Sequencing. J. Fungi (Basel) 7 (8), 636. doi: 10.3390/jof7080636 34436175 PMC8398552

[B38] Vandenberg O. Durand G. Hallin M. Diefenbach A. Gant V. Murray P.. (2020). Consolidation of Clinical Microbiology Laboratories and Introduction of Transformative Technologies. Clin. Microbiol. Rev. 33 (2), e00057-19. doi: 10.1128/cmr.00057-19 32102900 PMC7048017

[B39] Yang B. Wang J. Tao X. Wang D. (2019). Clinical Investigation on the Risk Factors for Prognosis in Patients With Septic Shock. Zhonghua Wei Zhong Bing Ji Jiu Yi Xue 31 (9), 1078–1082. doi: 10.3760/cma.j.issn.2095-4352.2019.09.004 31657328

[B40] Yan G. Liu J. Chen W. Chen Y. Cheng Y. Tao J.. (2021). Metagenomic Next-Generation Sequencing of Bloodstream Microbial Cell-Free Nucleic Acid in Children With Suspected Sepsis in Pediatric Intensive Care Unit. Front. Cell Infect. Microbiol. 11. doi: 10.3389/fcimb.2021.665226 PMC8421769 34504805

[B41] Zhang Y. Ai J. W. Cui P. Zhang W. H. Wu H. L. Ye M. Z.. (2019). A Cluster of Cases of Pneumocystis Pneumonia Identified by Shotgun Metagenomics Approach. J. Inf Secur 78, 158–169. doi: 10.1016/j.jinf.2018.08.013 30149030

[B42] Zhang H. C. Ai J. W. Cui P. Zhu Y. M. Wu H. L. Li Y. J.. (2019). Incremental Value of Metagenomic Next Generation Sequencing for the Diagnosis of Suspected Focal Infection in Adults. J. Infect. 79 (5), 419–425. doi: 10.1016/j.jinf.2019.08.012 31442461

[B43] Zhang F. Chen J. Huang H. Deng X. Zhang W. Zeng M.. (2012). Application of Metagenomic Next-Generation Sequencing in the Diagnosis and Treatment Guidance of Pneumocystis Jirovecii Pneumonia in Renal Transplant Recipients. Eur. J. Clin. Microbiol. Infect. Dis. 40 (9), 1933–1942. doi: 10.1007/s10096-021-04254-x PMC8057919 33880744

[B44] Zhan L. Huang K. Xia W. Chen J. Wang L. Lu J.. (2022). The Diagnosis of Severe Fever With Thrombocytopenia Syndrome Using Metagenomic Next-Generation Sequencing: Case Report and Literature Review. Infect. Drug Resist. 15, 83–89. doi: 10.2147/idr.S345991 35046673 PMC8760998

[B45] Zheng Y. H. Lin W. Zhang T. L. Fang Y. Chen B. W. Pan G. Q.. (2022). Value of Metagenomic Next-Generation Sequencing in Children With Severe Infectious Diseases. Zhongguo Dang Dai Er Ke Za Zhi 24 (3), 273–278. doi: 10.7499/j.issn.1008-8830.2110003 35351257 PMC8974643

[B46] Zhou H. Larkin P. M. K. Zhao D. Ma Q. Yao Y. Wu X.. (2021). Clinical Impact of Metagenomic Next-Generation Sequencing of Bronchoalveolar Lavage in the Diagnosis and Management of Pneumonia: A Multicenter Prospective Observational Study. J. Mol. Diagn. 23 (10), 1259–1268. doi: 10.1016/j.jmoldx.2021.06.007 34197923

